# Effects of differences in serum total homocysteine, folate, and vitamin B_12_ on cognitive impairment in stroke patients

**DOI:** 10.1186/s12883-014-0217-9

**Published:** 2014-11-30

**Authors:** Bo Jiang, Yumei Chen, Guoen Yao, Cunshan Yao, Hongmei Zhao, Xiangdong Jia, Yunyan Zhang, Junling Ge, Enchao Qiu, Chengyun Ding

**Affiliations:** Department of Neuromedical Center, The First Hospital Affiliated to the Chinese PLA General Hospital, 51 Fucheng Avenue, Beijing, 100048 Haidian District China

**Keywords:** Cognitive impairment, Cerebrovascular disorder, Neuropsychology, Event related potentials P300, Homocysteine

## Abstract

**Background:**

Vascular cognitive impairment-no dementia (VCIND) refers to the early or mild cognitive impairment induced by cerebral vascular injury. Research shows that serum total homocysteine (tHcy) level is an independent risk factor for cerebral vascular disease and may be closely related to cognitive function.Current studies on the tHcy level in VCIND patients are limited, and the relationship of tHcy with cognitive function remains unclear. This study aims to investigate the tHcy levels in patients with VCIND and to determine their correlation with cognitive function, as well as to provide useful clues for preventing and treating VCIND.

**Methods:**

The tHcy, folate, and vitamin B_12_ levels in 82 patients with VCIND were reviewed retrospectively and compared with those of 80 stroke patients without cognitive impairment and 69 healthy controls by using the Montreal Cognitive Assessment (MoCA) scale and the event-related potential P300 to evaluate cognitive function.

**Results:**

The tHcy levels in the VCIND group were higher than those in the other two groups, whereas the folate and Vitamin B_12_ levels in the VCIND group were lower than those of the other two groups. The tHcy levels in the stroke group were higher than those in the control group, and the folate and vitamin B_12_ levels in the stroke group were lower than those in the control group. The patients in the VCIND group with high tHcy exhibited lower MoCA scores and prolonged P300 latency than those in with normal tHcy. Correlation analysis showed that tHcy level is positively correlated with P300 latency period and negatively correlated with MoCA score.

**Conclusion:**

The tHcy levels were significantly higher and the vitamin B_12_ and folate levels were significantly lower in the patients with VCIND than those in the other groups. The high tHcy levels in the VCIND patients may be correlated with impaired cognitive function.

## Background

Vascular cognitive impairment-no dementia (VCIND) refers to the early or mild cognitive impairment induced by cerebral vascular injury. The illness is relatively hidden, and the degree of cognitive impairment has not yet reached the diagnostic standard for dementia [1]. VCIND has an incidence of 39.5% within 1 year after a stroke [2]. Early diagnosis and intervention improve the prognosis of VCIND patients, which would otherwise progress into dementia [3]. Considering its reversibility, VCIND has become a hot research topic. Research shows that serum total homocysteine (tHcy) level is an independent risk factor for cerebral vascular disease [4] and may be closely related to cognitive function [5]. Current studies on the tHcy level in VCIND patients are limited, and the relationship of tHcy with cognitive function remains unclear. This study aims to investigate the tHcy levels in patients with VCIND and to determine their correlation with cognitive function, as well as to provide useful clues for preventing and treating VCIND.

### Participants and methods

#### Participants

From January 2008 to January 2013, 367 new stroke patients were screened from the Department of Internal Medicine of the First Affiliated Hospital of PLA General Hospital. All the stroke patients performed the cognitive function detection on the post-onset seventh day, first month, and third month. The patient would be enrolled in the study when the VCIND inclusion criteria were met. The detection of related indicators such as folate, vitamin B_12_, tHcy, and P300 were completed within 48 h of enrollment. Among 97 patients that met the VCIND inclusion criteria, 5 refused to join the experiment, and 10 were not enrolled because they had severe internal diseases or took medication that would affect tHcy. Finally, 82 VCIND patients were recruited. Currently, there are no unified diagnostic criteria of VCIND, and different studies might use different diagnostic criteria [3,6]. In our study, VCIND was diagnosed according to the Rockwood criteria [7] as follows: existing cerebrovascular disease; evidence of cognitive impairment under neuropsychological assessment; the cognitive impairment occurred within 3 months after stroke; causal relationship between cerebrovascular disease and cognitive impairment, excluding other diseases; Hanchinski ischemia index ≥7; does not conform to the diagnostic criteria for dementia revised by the United States of America Diagnostic and Statistical Manual of Mental Disorders, Fourth Edition (DSM-IV). The exclusion criteria were as follows: 1) Alzheimer’s patients; 2) other cognitive disorders, mental illness, or hemiplegic aphasia and other diseases that might influence their Montreal score and P300 determination; 3) taking medications that affect tHcy levels within the past 1 month (such as contraceptives, anti-epileptic drugs, dopamine drugs, and folate and/or vitamin B_12_); 4) the presence of diseases that affect central nervous system function, such as thyroid disease, severe anemia, vitamin B_12_ and folate deficiency, and severe malnutrition, as well as serious liver, kidney, and other organic diseases.

A total of 80 outpatient and hospitalized stroke patients were enrolled in the study. Stroke was diagnosed in accordance with the diagnostic criteria [8], revised by the 2003 European Stroke Promotion Association, and was confirmed by MRI scanning and scale detection, as well as via clinical and cognitive function detection without cognitive impairment.

The control group consisted of 69 healthy controls who were also in the First Affiliated Hospital of PLA General Hospital. The cranial MRI showed no obvious lesion, and the clinical and cognitive function determination did not found obvious impairment. This study was conducted in accordance with the declaration of Helsinki, and was conducted with approval from the Ethics Committee of the First Hospital Affiliated to the Chinese PLA General Hospital. Written informed consent was obtained from all participants.

## Methods

### tHcy, folate, and vitamin B_12_ detection

Fasting venous blood samples (2–3 mL), which were centrifuged to obtain serum after 30 min, were drawn from each subject. The tHcy concentration was determined via enzymatic conversion on a Hitachi 7180 automatic biochemical analyzer (Tokyo, Japan) using a kit provided by Beijing Nine Strong Biological Technology Co. Ltd. (Beijing, China). The tHcy levels ranged from 5 to 14 μmol/L. Hyperhomocysteinemia (Hhcy) was defined as a level >14 μmol/L. Vitamin B_12_ and folate levels were determined from 3 to 4 mL of venous blood using an Access automated chemiluminescent microparticle immunoassay system and the related kit (BECKMAN Company USA).

### Cognitive function tests

Cognitive function was evaluated using the Montreal Cognitive Assessment scale (MoCA) [9]. The MoCA includes eight cognitive domains and eleven checking contents, including visuospatial ability, naming, memory, attention and calculation, language fluency, abstract thinking, delayed memory, and directional force. The highest possible score is 30 points. Participants who had less than 12 years of schooling were given an additional point in their final score. Higher scores indicate better cognitive function. The impairment assessment criterion was a MoCA <26.

### P300 potential measurement

All of the patients underwent a P300 determination within 48 h by using the British Oxford multimedia EMG/EP system. In a quiet, shielded room, the participants were instructed to lie in a supine position, stay awake, and concentrate. In accordance with International EEG 10–20 system, recording electrodes were placed in the central line. The reference electrode was placed on the right lobe, with frontal grounding. The inter electrode impedance was <5 KΩ, and the analysis time was 600 ms. Using Tone Pip stimuli, the probability of the non-target stimuli (1000 Hz) was set to 80% with a magnitude of 80 dB with gradations. The target stimulus (4000 Hz) was set to 20% probability with a magnitude of 90 dB and was interspersed randomly with the non-target stimuli. The participants were instructed to respond to the target stimulus by knobbing the key. The instrument automatically recorded the reaction time. The test was repeated twice, and the mean score was used in the data analysis.

### Statistical analysis

Data were analyzed using SPSS16.0 software. The recorded data are expressed as mean ± SD and were compared using an analysis of variance and a *t*-test. Nonparametric Mann–Whitney U tests were performed on the level of the variable. Count data are expressed as percentages and compared using a χ^2^ test. Correlation analysis was performed via Pearson correlation analysis. Differences with P <0.05 were considered significant.

## Results

### Characteristics of the participants

Among the 82 VCIND patients, 73 were inpatients and 9 were outpatients, 49 were male and 33 were female, and were 41–78 years old, with a mean age of 64 ± 3.14 years. The educational background of the patients varied, with 14 cases reaching university, 57 cases reaching high school, and 11 cases reaching junior high school. The National Institutes of Health Stroke Scale (NIHSS) score was 5.17 ± 1.45, including 35 diabetic, 41 hypertensive, and 29 hyperlipidemic patients.

Of the 80 stroke patients, 51 were male and 29 were female, with ages ranging from 37 to 77 years and a mean age of 62 ± 2.79 years. The educational status of the patients varied as follows: 13 received a university education, 53 reached high school, and 14 reached junior high school. Their NIHSS score was 4.77 ± 1.79, including those of 31 diabetic, 37 hypertensive, and 27 hyperlipidemic patients.

The control group consisted of 42 males and 27 females and included 11 diabetic, 16 hypertensive, and 15 hyperlipidemic patients. Their ages ranged from 41 to 72 years, with a mean age of 61 ± 1.89 years. Their educational backgrounds were as follows: 17 received a university education, 45 reached high school, and 7 reached junior high school.

### Comparison of general states

The three groups did not differ significantly differ in terms of age (based on the ANOVA, P >0.05), gender, culture level, and the proportion of patients (based on the χ^2^, P >0.05). The VCIND group did not differ from the stroke group in terms of NIHSS (Mann–Whitney U test, P >0.05). The VCIND group did not differ significantly from the stroke group in terms of the incidence of diabetes, hypertension, and hyperlipidemia (χ^2^, P >0.05).

### Cognitive function

The VCIND group had significantly lower MoCA scores (22.81 ± 1.67) than did the other two groups (P <0.01). The stroke group (27.77 ± 1.03) did not differ significantly from the control group (28.23 ± 0.91) in terms of the total MoCA score (P >0.05).

### tHcy, folate, and vitamin B_12_

The tHcy level in the VCIND group was higher than that of the other two groups. The folate and the vitamin B_12_ levels in the VCIND group were lower than those in the other two groups. The tHcy level in the stroke group was higher than that in the control group, and the folate and vitamin B_12_ levels were lower than those in the control group (Table 1).

**Table 1:** **Comparison of serum tHcy (μmol/L), folate (ng/ml), Vitamin B**_12_ **(pg/ml) level between the three groups** $\left(\overline{\times} \pm \mathbf{s}\right)$

**Group**	**Cases (n)**	**tHcy**	**Folate**	**Vitmin B**_**12**_
VCIND	82	22.14 ± 6.92^a^	8.01 ± 3.13^a^	280.85 ± 96.72^b^
Stroke	80	16.36 ± 7.17^d^	12.61 ± 3.56^c^	367.53 ± 127.30^c^
Control	69	11.86 ± 4.47	16.42 ± 4.91	495.18 ± 102.79
F		13.36	22.81	20.14
*P*		<0.001	<0.001	<0.001

Note: Compared with the other two groups: ^a^ *P* < 0.01, ^b^ *P* < 0.05; compared with the control group, ^c^ *P* < 0.01, ^d^ *P* < 0.05.

### Determination of P300

The prolongation of the P300 latency period in the VCIND group was significantly longer than that in the stroke and control groups (P <0.01). The prolongation in the P300 latency period in the stroke group did not differ significantly from that in the control group (P >0.05). The three groups did not differ significantly in terms of P300 amplitude (P >0.05), which is shown in Table 2.

**Table 2:** **Comparison of the central midline P300 latency period(MS) and amplitude (μV) between the three groups** $\left(\ \overline{\times} \pm \mathrm{s}\right)$

**Group**	**Cases (n)**	**P300 latency**	**P300 amplitude**
VCIND	82	396.68 ± 76.67^a^	3.86 ± 0.87
Stroke	80	307.20 ± 50.45	3.80 ± 0.78
Control	69	306.13 ± 70.33	3.83 ± 0.69
F		12.04	0.028
*P*		0.028	0.973

Note: Compared with the other group, ^a^ *P* < 0.01.

### Comparison of MoCA and P300 in the VCIND group

In the VCIND group, 45 patients exhibited Hhcy. The patients with Hhcy had lower MoCA scores and prolongation in the P300 latency period than the patients with normal tHcy, but the P300 amplitude did not differ significantly between the groups (Table 3).

**Table 3:** **Comparison of MoCA, P300 between the Hhcy (tHcy > 14 μmol/L) group and normal tHcy group in VCIND group** $\left(\ \overline{\times} \pm \mathrm{s}\right)$

**Groups**	**Cases (n)**	**MoCA**	**P300 latency**	**P300 amplitude**
Normal tHcy	37	23.71 ± 0.91^a^	371.67 ± 31.79^a^	3.88 ± 0.60
Hhcy	45	21.53 ± 1.36	429.07 ± 41.87	3.73 ± 0.78
*t*		5.041	4.229	0.561
*P*		<0.001	<0.001	>0.05

Note: ^a^ *P* < 0.01.

### Correlation between the tHcy, folate, vitamin B_12_, P300 latency period, and MoCA in the VCIND group

The tHcy levels in the VCIND group patients were correlated with their P300 latency periods and MoCA scores. The tHcy levels were negatively correlated with MoCA score (r = −0.468, P = 0.038) and were positively correlated with the P300 latency period (r = 0.740, P = 0.014). The folate level was positively correlated with MoCA score (r = 0.509, P = 0.022), and the vitamin B_12_ level was positively correlated with MoCA score (r = 0.588, P = 0.006).

## Discussion

tHcy is a thiol-containing amino acid generated from methionine through in vivo metabolism [10]. Many studies have shown that high tHcy levels are related to cognitive function damage [11-13]. The influence of tHcy on the cognitive functions of VCIND patients has important clinical significance.

The tHcy levels in the VCIND and stroke groups were higher than in the normal group. This finding suggests that tHcy levels are correlated with the pathogenesis of stroke and is consistent with the current research results. Specifically, tHcy is an independent risk factor for cerebral vascular disease [14,15]. The tHcy levels in the VCIND group were higher than those in the stroke group, which suggests that cognitive function may be correlated with tHcy levels.

Previous studies on the relationship between tHcy and cognitive function differ in their findings [16-18]. Different ethnic groups have different apolipoprotein E (ApoE) genes, which may be associated with cognitive function [19]. Currently, it is considered that the cognitive impairment caused by tHcy occurs through direct and indirect paths; the direct path involves increasing glutamate excitotoxicity, thereby reducing neuronal DNA repair capacity and accelerating the formation of oxidative stress and A β and damage on hippocampal neurons, which leads to cognitive impairment. The indirect path involves vascular endothelial cell dysfunction and lipid metabolic disorder, leading to cerebral vascular disease to cause impairment of cognitive function [20-22]. A certain randomized, double-blind clinical trial showed that reduced tHcy could not necessarily improve cognitive functions [23,24] and, thus, suggested that the current mechanism of the damages of cognitive functions caused by tHcy was not entirely clear.

The patients in the VCIND group exhibited higher tHcy levels than those in the other two groups. Within the VCIND group, the MoCA score of the patients with high tHcy was lower than those with normal tHcy. Furthermore, correlation analysis shows that tHcy level is negatively correlated with MoCA, which suggests that tHcy levels may be closely associated with impaired cognitive function.

The P300 event-related potential is an objective electrophysiologic examination that reflects brain cognition function [25,26]. Related research has shown that patients with mild cognitive impairment exhibit changes in P300 [27]. The P300 latency period reflects the evaluation time of stimuli in cognitive activities, which is an index for information processing speed [28], reflecting to a certain degree the perception, attention, memory, information coding, and cognitive integration speed brain function state [29]. The results of this study show that the P300 latency period in the VCIND group was significantly longer than in the other two groups, which indicates stable and high sensitivity of the P300 latency period and the abnormalities during mild cognitive impairment. Correlation analysis shows that the tHcy level in the VCIND group is positively correlated with the P300 latency period, which further supports the association of tHcy levels with impaired cognition.

## Conclusions

VCIND patients have significantly higher levels of tHcy and lower levels of vitamin B_12_ and folate. The increased tHcy in VCIND patients may be related to cognitive impairment, which provides a possibility for the treatment of tHcy levels to improve cognitive function in VCIND patients, which should be confirmed in future research work.

This study mainly selected the elderly as the research subjects, and the sample size was relatively small, which might be the limitation of this study. This study also did not consider the effect of folate and vitamin B_12_ directly on cognitive function. Therefore, we recommended increasing the number of cases and the statistical processing methods in future studies for a more reliable conclusion.
